# Reemergence of human plague in Yunnan, China in 2016

**DOI:** 10.1371/journal.pone.0198067

**Published:** 2018-06-13

**Authors:** Liyuan Shi, Guirong Yang, Zhikai Zhang, Lianxu Xia, Ying Liang, Hongli Tan, Jinrong He, Jianguo Xu, Zhizhong Song, Wei Li, Peng Wang

**Affiliations:** 1 Yunnan Institute for Endemic Disease Control and Prevention, Yunnan, China; 2 Yunnan Provincial Key Laboratory for Zoonosis Control and Prevention, Yunnan, China; 3 National Institute for Communicable Disease Control and Prevention, Chinese Center for Disease control and Prevention, Changping, Beijing, China; 4 State Key Laboratory of Infectious Disease Prevention and Control, Beijing, China; 5 Collaborative Innovation Center for Diagnosis and Treatment of Infectious Disease, Zhejiang, China; University of Minnesota, UNITED STATES

## Abstract

The third plague pandemic originated from Yunnan Province, China in the middle of the 19^th^ century. The last human plague epidemic in Yunnan occurred from 1986–2005. On June 6, 2016, a case of human plague was reported in the Xishuangbanna Prefecture, Yunnan. The patient suffered from primary septicemic plague after exposure to a dead house rat (*Rattus flavipectus*), which has been identified as the main plague reservoir in the local epizootic area. Moreover, a retrospective investigation identified another bubonic plague case in this area. Based on these data, human plague reemerged after a silent period of ten years. In this study, three molecular typing methods, including a clustered regularly interspaced short palindromic repeats (CRISPR) analysis, different region analysis (DFR), and multiple-locus variable number of tandem repeats analysis (MLVA), were used to illustrate the molecular characteristics of *Yersinia pestis* (*Y*. *pestis*) strains isolated in Yunnan. The DFR profiles of the strains isolated in Yunnan in 2016 were the same as the strains that had previously been isolated in this *Rattus flavipectus* plague focus. The c3 spacer present in the previously isolated strains was absent in the spacer arrays of the Ypc CRISPR loci of the strains isolated in 2016. The MLVA analysis using MLVA (14+12) showed that the strains isolated from the human plague case and host animal plague infection in 2016 in Yunnan displayed different molecular patterns than the strains that had previously been isolated from Yunnan and adjacent provinces.

## Introduction

Plague is an acute infectious disease caused by *Yersinia pestis* (*Y*. *pestis*). Plague killed millions of individuals in Europe in the 14^th^ century. In China, tens of thousands of individuals died of plague in the 19^th^ century [1]. Plague is primarily a disease carried by rodents and is transmitted among animals bitten by infected fleas. Cases of human plague occur when these fleas, notably *Xenopsylla cheopis*, bite humans. Outbreaks of human plague frequently occur as a result of epizootics in rats, particularly rat species that have flourished around human habitations, such as *Rattus rattus* and *Rattus flavipectus*.

Three major types of plague exist in humans: bubonic, pneumonic and septicemic plague. Person-to-person transmission typically occurs only among patients with pneumonic plague. Two subspecies of the pathogens that cause plague have been recognized based on their pathogenicity in the anthroponotic form: *pestis* and *microtus* [2]. Four biovars have also been classified according to their biochemical properties and include antiqua, mediaevalis, orientalis and microtus [3]. These biovars have been implicated in several devastating pandemics over the previous 1500 years, with the exception of the microtus biovar [1]. The third plague pandemic was caused by *Y*. *pestis* biovar orientalis (positive for nitrate reduction and negative for glycerol utilization) and originated from Yunnan Province in China in the middle of the 19^th^ century; it eventually affected more than 60 countries and regions worldwide [1].

Based on historical data, Yunnan Province was seriously affected by the plague prior to 1955 [4]. Currently, three types of plague foci are detected in Yunnan Province, namely, the Yunnan *Rattus flavipectus* plague focus (also regarded as the house rat plague focus by Chinese scientists, as rodent hosts have been determined to mainly inhabit local residents’ houses or yards). The other two types include Jianchuan and Yulong plague foci (these two foci have been characterized as wild mouse (sylvatic) plague foci by Chinese plague researchers). The main reservoir and vector in the Yunnan house rat plague focus (Urban rat plague focus) are the *R*. *flavipectus* and the *Xenopsylla cheopis*. Together with the Guangdong and Fujian provinces’ house rat plague foci identified in the 1950s, these plague foci have been identified as *R*. *flavipectus* plague foci in China, and the *Y*. *pestis* biovars are orientalis.

In China, plague epidemics have been successfully controlled in house rat foci since the mid-1950s. Moreover, active animal plague surveillance programs have been conducted annually in China since the 1980s. However, after a 26-year silent period, a human plague epidemic reemerged in the western region in Yunnan and then spread eastward from 1986–2005 [5]. The areas that were most substantially affected by this epidemic are located in the southern and southwestern regions of Yunnan Province. Five hundred seven human cases and two deaths were confirmed during this plague epidemic. Twenty-nine of the 129 counties and nine of the 16 prefectures (cities) were affected [5]. This epidemic was also caused by *Y*. *pestis* biovar orientalis [5]. Adjacent provinces, such as Guizhou and Guangxi, were affected in 2000–2002. The human plague epidemic and rodent plague epizootic ended in 2005 and 2007, respectively. However, a human plague infection with characteristics of *Y*. *pestis* biovar orientalis strains was reported in 2016 in Yunnan. In the present study, three molecular subtyping methods, including different region analysis (DFR), clustered regularly interspaced short palindromic repeats (CRISPR), and multiple-locus variable number of tandem repeats analysis (MLVA), were used to describe the genetic relationships among the strains isolated in 2016, as well as isolates from the previous plague epidemics in the house rat plague focus. In addition, a source tracing investigation was performed using the MLVA(14+12) scheme.

## Methods

### Ethics statement

All procedures involved in the survey and diagnosis of human plague cases were performed in accordance with ethical standards. This research was approved by the Ethical Committee [Institutional Review Board (IRB)] of National Institute for Communicable Disease Control and Prevention (ICDC), China CDC (License number: ICDC-2014013). All animal sampling procedures conducted in this study involved only three dead rats; thus, animals were not sacrificed in the present study. The procedures were conducted in accordance with the animal care and use regulations of the Institute for Communicable Disease Control and Prevention (ICDC).

### Epidemiological investigation of human plague in Yunnan in 2016

On May 28, 2016, a suspected case of human plague in Jinghong County of the Xishuangbanna (subsequently referred to as Banna) Prefecture (S1 Fig) was reported to the China National Health and Family Planning Commission. Enhanced surveillance of rats in the habitations of local residents was subsequently conducted in this region. Gram staining, bacteriophage lysis testing, polymerase chain reaction (PCR), a reverse indirect hemagglutination assay (RIHA) and a colloidal gold-immunochromatography assay were used to isolate and identify bacteria present in the collected samples. Biochemical assays, including arabinose fermentation, glycerol fermentation, and nitrate reduction, were also performed for further identification. The antibody test against the F1 antigen via an indirect hemagglutination assay (IHA) [6] was used to identify one retrospective bubonic plague case in an epidemiological investigation.

### Strains included in this study

**Fig 1:**
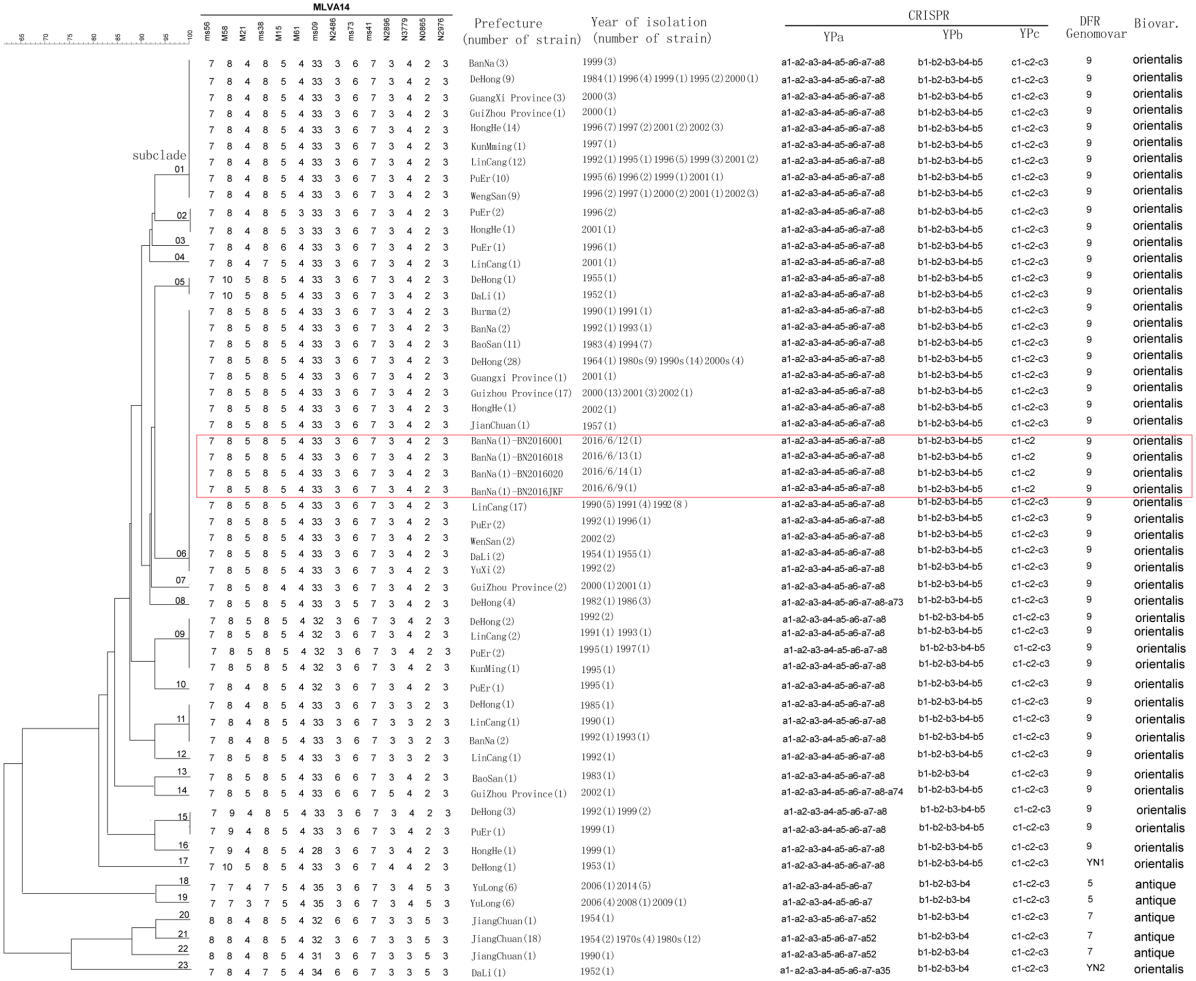
Dendrogram of the *Y*. *pestis* strains isolated in Yunnan obtained using the MLVA14 scheme. The number on the dendrogram line indicates the MLVA14 scheme subclade.

Two hundred twenty-one *Y*. *pestis* strains isolated from 1952 to 2016 in Yunnan and adjacent provinces (Guizhou and Guangxi) were included in this study, as well as four *Y*. *pestis* isolates obtained in Yunnan in 2016 (one from a patient and three from dead rats collected near the patient’s house). In addition, representative sylvatic plague strains (biovar antiqua) from the Jianchuan and Yulong plague foci in Yunnan Province were also included in this study (Fig 1). All strains were collected from the Yunnan Institute for Endemic Disease Control and Prevention, Centers for Disease Control and Prevention of Guizhou Province and Guangxi Province, and National Institute for Communicable Disease Control and Prevention (ICDC), China CDC. The bacteria were cultivated in nutrient agar at 28°C for 48 hours, and then the genomic DNA was extracted from each bacterium using the QIAGEN DNeasy blood & tissue kit (QIAGEN Shanghai, China) in a Biosafety Level 3 (BSL-3) Laboratory of the ICDC according to the manufacturer’s instructions. The chromosomal DNA was diluted 1:10 with sterile nuclease-free H_2_O and was used as a template for PCR amplification.

### Molecular subtyping using DFR, CRISPR, and MLVA

The CRISPR analysis [3] and DFR genotyping analysis [7–9] were performed as previously described in the literature. The CRISPR spacer arrays utilized in the present study were obtained from the “spacer dictionary” [3] or analyzed online using the “CRISPR Finder Tool” in the CRISPR database [10]. Twenty-three DFR and *pMT*1-specific primers were used to identify DFR loci. The nomenclatures of the spacer arrays and DFR genomovars were employed in a manner consistent with previous publications [7]. An MLVA analysis with 26 markers (14+12) was performed using the methods described by Li et al. [11].

### Genetic source tracking in 2016

Bionumerics 6.6 software (Applied-Math, Belgium) was used to conduct the DFR, CRISPR, and MLVA(14+12) molecular subtyping analyses. DFR, CRISPR, and MLVA14 were used to illustrate the phylogenetic relationships among isolates collected in Yunnan and adjacent provinces. The MLVA(14+12) scheme was used for genetic source tracking among the strains grouped into the same subclades with the human plague case-related strains isolated in 2016. The MLVA profiles were analyzed as categorical data and the UPGMA (unweighted pair group method using arithmetic averages) was used as a clustering arithmetical algorithm. Corresponding dendrograms or minimum spanning trees (MSTs) were constructed using the neighbor-joining method with the Dice genetic distance coefficient [11].

## Results

### Human plague occurred in Yunnan in 2016

On May 28, 2016, a human plague case was identified in a 68-year-old woman who had no underlying diseases. The patient lived in Jinghong County of the Banna Prefecture (S1 Fig), which is adjacent to Burma and Laos. On June 1, 2016, the patient was admitted to the local hospital with a high fever (39.8°C), weakness and a slight cough. Lymphadenopathy was not observed. The results of the biochemical analyses conducted in the local hospital suggested that a *Y*. *pestis* infection could not be excluded due to the presence of *Y*. *pestis* in the blood (probability rate 99%). This human plague case was confirmed via PCR targeting the *caf1* and *YPO0392* genes of *Y*. *pestis*. The bacteria isolated from the patient were then specifically identified as *Y*. *pestis* by gram staining, microscopy, and a phage lysis test. The results of other laboratory tests, including RIHA and colloidal gold-immunochromatography assay, indicated that the F1 antigen was present in the blood sample. Serum IHA titers for antibodies against the F1 antigen were 1:10 (on June 6, 2016), 1:80 (on June 9, 2016), and 1:320 (on June 12, 2016); the patient was ultimately diagnosed with primary septicemic plague by the local and provincial CDCs because she did not exhibit symptoms of lymphadenopathy. Antibiotic therapy was administered through intravenous injections of 2 g of Cefpiramide (twice daily) initiated on June 2, 2016 and intravenous injections of 100 ml of Etimicin (twice daily) initiated on June 12, 2016. The patient was clinically cured on June 18, 2016. Fifty-eight individuals who had close contact with the patient were placed under quarantine and medical observation. None of the contacts had symptoms of the disease by the end of the nine-day quarantine. Through a retrospective investigation, another human plague case (a 55-year-old man) with lymphadenopathy was identified. This bubonic plague case was first identified on May 15, 2016, and the patient had been cured with antibiotic therapy by the time of our investigation. According to the IHA results, the serum sample from this patient was positive for the F1 antibody (titer 1:160). The biochemical analysis indicated that the strains isolated from the female patient and three isolates from local reservoirs (*R*. *flavipectus)* obtained during routine animal plague surveillance were biovar orientalis.

### Molecular subtyping using DFR, CRISPR and MLVA14

The DFR, CRISPR method and an MLVA14 scheme were used to illustrate the phylogenetic relationships among biovar orientalis isolates from house rat plague foci and two sylvatic plague foci (biovar antiqua) in China. The DFR genomovars of the four orientalis isolates obtained in 2016 were genomovar 09 (Fig 1). The *Y*. *pestis* biovar orientalis strains isolated in Yunnan exhibited highly homologous DFR patterns, e.g., in the majority of the strains, genomovar 09 was predominant, with the exception of two strains that belonged to new DFR genomovars (referred to as YN1 and YN2 and in which DFR10 and DFR9, respectively, were absent). The corresponding functions of the *DFR10* and *DFR9* genes have been annotated as an outer membrane protease and prophage, respectively [9].

The arrays of the CRISPR loci identified in the *Y*. *pestis* strains isolated from the human plague case and from dead host animals in local epizootics in 2016 exhibited special space patterns, i.e., Ypa (a1-a2-a3-a4-a5-a6-a7-a8), Ypb (b1-b2-b3-b4-a5), and Ypc (c1-c2) (Fig 1). The CRISPR patterns in the *Y*. *pestis* biovar orientalis strains that had previously been isolated from rodents and vectors in Yunnan or adjacent provinces belonged to genotypes 30 or 33 in the Ca8 cluster, i.e., Ypa (a1-a2-a3-a4-a5-a6-a7-a8) or Ypa (a1-a2-a3-a4-a5-a6-a7-a8-a73), Ypb (b1-b2-b3-b4-b5), and Ypc (c1-c2-c3). Thus, the Ypa and Ypb loci in the 2016 isolates were the same as the strains in genotype 30 in the Ca8 cluster, whereas the c3 spacer was absent in the spacer arrays of the Ypc CRISPR loci of the strains isolated in 2016. These patterns have not been previously identified among *Y*. *pestis* strains isolated in China or other previous publications [3,12–16].

**Fig 2:**
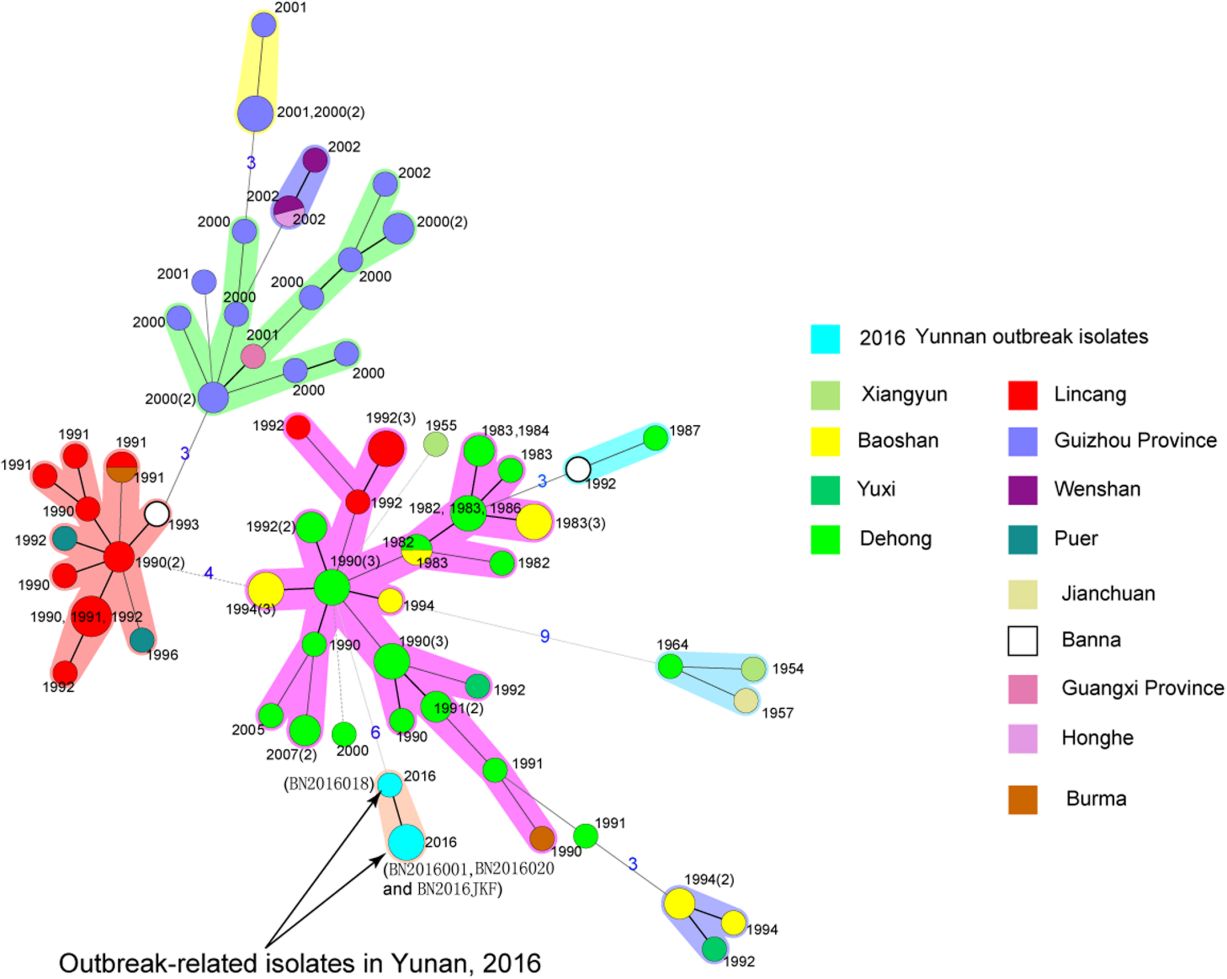
Minimum spanning tree (MST) of the isolates in the MLVA14-06 subclade obtained using the MLVA(14+12) scheme. Thick solid lines connect types that differed in a single VNTR locus, thin solid lines connect types that differed in 2 VNTR loci, and other lines connect types that differed in more than 2 VNTR loci. Circles with different colors indicate prefectures in Yunnan or other provinces or strains isolated in Burma. The year in which the isolate was obtained is indicated by the lineages. The numbers on the branches indicate the different numbers of VNTR loci. If two neighboring types did not differ in more than 2 VNTR loci, they would be surrounded by a halo of the same color.

The MLVA14 scheme revealed 23 subtypes among the strains analyzed in the present study (Fig 1). Two dominant subtypes of MLVA14, which account for 81.4% (154/189) of the biovar orientalis isolates detected in the present study, were identified as the MLVA14-01 subclade (or the 01 subclade within the MLVA14 scheme) and the MLVA14-06 subclade; the abovementioned subclades accounted for 32.8% and 48.7% of biovar orientalis isolates, respectively. Thus, two main *Y*. *pestis* clones were presumed to be present in the Yunnan plague epidemics during 1982 and 2006. The strains implicated in the more recent human plague and host animals plague epizootics in 2016 also fell into the MLVA14-06 subclade; other strains in the MLVA14-06 subclade included strains isolated from 9 prefectures in Yunnan Province (n = 68), Guizhou Province (n = 17), Guangxi Province (n = 1), and Burma (n = 2) in different years (Figs 1 and 2). Furthermore, two strains isolated from the Banna Prefecture in 1992 and 1993 were clustered together with human plague strains isolated in 2016. In addition, one orientalis isolate collected in 1957 and one isolate collected in 1964 also fell into the MLVA14-06 subclade (Fig 1).

### Source tracing of the related strains in 2016 using MLVA(14+12) analysis

Based on the results of the MLVA14 scheme, twelve variable number of tandem repeats (VNTRs) were further complementally used for source tracing of the related strains identified in Yunnan in 2016. An MST was constructed to describe the diversity of the *Y*. *pestis* biovar orientalis in the MLVA14-06 subclade (Fig 2). The corresponding dendrogram of the MLVA(14+12) scheme for isolates in the MLVA14-06 subclade is presented in S2 Fig. According to the results of the epidemiological investigation in 2016, a plague epizootic occurred in local areas, and three *Y*. *pestis* isolates from three dead rats (*R*. *flavipectus*) were obtained in the epizootic. Two strains isolated from two dead rats in the 2016 outbreak were clustered together with the patient’s strain using the MLVA(14+12) scheme, whereas only one VNTR locus among the 26 VNTR loci differed between the isolate from the third dead rat and the isolate from the human case. Therefore, the strain isolated from the patient was transmitted by infected rats. No other strain in the MLVA14-06 subclade was homologous to the isolates collected in 2016 (Fig 2). In general, the isolates associated with the reemergence of human plague in 2016 differed from the isolates that had previously been collected in the biovar orientalis plague epidemics.

## Discussion

Yunnan Province borders Burma, Laos, and Vietnam, and it is also adjacent to the Guizhou, Guangxi, Tibet and Sichuan provinces in southwestern China. In the Yunnan *R*. *flavipectus* plague focus, no cases of human plague had been reported since 1955. However, after a 26-year silent period, a human plague epidemic swept from western to eastern regions in Yunnan between 1986 and 2005 [5]. In fact, the presence of a plague epizootic was observed in 1982 [17]. More intensive rodents plague surveillance has been performed in Yunnan since the human plague epidemic began in 1986. No rodent plague epidemics were observed in house rat plague foci (Urban rat plague focus). Moreover, no human plague cases were reported for this focus after the end of the previously described epidemic; this state lasted for one decade until two human plague cases were reported in 2016 [5]. The occurrence of two human plague cases in 2016 suggests a reemergence of human plague caused by *Y*. *pestis* biovar orientalis strains in China; corresponding phylogenetic evidence supports the conclusion that human plague cases identified in Yunnan in 2016 originated from rats that died of plague.

In epidemiological investigations, the use of reliable subtyping methods is a prerequisite for identifying links among isolates and for understanding the dynamics of pathogen spread. Different molecular genotyping methods based on different principles have different applications. For example, the DFR analysis, which is mainly based on horizontal gene transfer or gene loss [7–9], has been used to illustrate the microevolution of *Y*. *pestis*. The spacer sequences in CRISPR loci are considered an immunological mechanism against bacteriophages or plasmids and might participate in cellular regulatory mechanisms [18]. Therefore, the spacer arrays of CRISPR loci may serve as a “chronicle” that documents the occurrence of the previously described bacterial gene events and may be used to illustrate the adaptive microevolution of *Y*. *pestis* [3]. The strain isolated from patients in the 2016 case shared a similar DFR profile with the strains previously isolated in Yunnan; however, the c3 spacer was absent in the spacer arrays of the Ypc CRISPR loci of the strains isolated in 2016. These CRISPR patterns had not been observed in the *Y*. *pestis* strains that were previously isolated in Yunnan or reported in the available literature [3,12–16].

A notable advantage of MLVA typing is its flexibility. Different sets of VNTR loci may be used to assess the performance of different phylogenetic investigations over different temporal or spatial scales. Previously described MLVA methods included 25 and 42–46 VNTR loci [19–22]. Using a serial hierarchical assessment, an optimized 14 VNTR loci-based scheme has been developed with a corresponding discriminatory power that may be considered compatible with single nucleotide polymorphism-based phylogenic analysis [11], whereas the inclusion of an additional twelve loci with greater diversity may be useful when tracking the source of the outbreak. The phylogenetic relationship yielded by the MLVA14 scheme indicated that some strains isolated in previous epidemics in the southern provinces of China (Yunnan, Guizhou, and Guangxi), including strains isolated in Burma, exhibited homologous MLVA14 profiles with the isolates collected in 2016 (Fig 1). Thus, based on the MLVA14 data, the pathogen that caused the human case in Yunnan in 2016 was similar to the pathogens responsible for previous epidemics in Yunnan or other regions.

Some researchers have investigated the possible mechanism of the interepizootic persistence of plague, such as the persistence of *Y*. *pestis* in soil, where plague microbes may be preserved in microfoci, etc. [23]. In fact, the mechanisms of interepizootic maintenance of plague are still a main question that remains unanswered. Many factors, including biotic (ecological) and abiotic (environmental: humidity, soil chemistry, etc.), influence *Y*. *pestis* and plague dynamics [23]. In fact, any ecological disturbance that changes the dynamics of the main host or main vector, even human activities, such as agricultural activities or a mass slaughter of the rodents, could unleash a flood of homeless fleas that would then transfer to humans and domestic animals and infect them with plague.

Interestingly, the cases of human plague that occurred in 2016 had a close relationship with the strains isolated in Dehong and Baoshan during the last *Y*. *pestis* biovar orientalis epidemic. Therefore, we hypothesized that the 2016 plague event in Yunnan was the successor of the previous epidemic, although several genomic differences were observed, such as the absence of the c3 spacer identified by the CRISPR analysis and the diversity of the 12 VNTRs identified by the MLVA(14+12) scheme. We also want to emphasize that *Y*. *pestis* may be transported from comparatively distant areas by rodents. Therefore, we were not able to exclude the possibility that *Y*. *pestis* may have been imported into the affected region from neighboring areas. More detailed investigations, including ongoing biological and anthropologic investigations, are necessary, and genome-based analyses may also provide additional information regarding the origin and microevolution of this bacterium.

The third plague pandemic caused by *Y*. *pestis* biovar orientalis originated from Yunnan Province in China in the middle of the 19^th^ century and killed millions of individuals in Asia, Europe, America, Africa and Australia. The human plague cases identified in Yunnan in 2016 suggests that *Y*. *pestis* biovar orientalis plague reemerged in China. Researchers should focus on the risks associated with animal or human plague, and an enhanced surveillance should be implemented in this area.

## Supporting information

S1 Fig Region of human plague occurrence in Yunnan in 2016. Upper left: Three plague foci in Yunnan; Lower left: the Geographic location (Xishuangbanna Prefecture) of human plague occurrence in 2016; Right: human plague occurrence region (Puwen town) in 2016.
(TIF)

S2 Fig Dendrogram of the *Y*. *pesti*s isolates in the MLVA14-06 subclade obtained using the MLVA(14+12) scheme. The columns depicted in different colors indicate prefectures in Yunnan or other provinces or strains isolated in Burma (the same strains as depicted in Fig 2).
(TIF)
