# Supplementary figures and images for: Reemergence of human plague in Yunnan, China in 2016
Source: PLoS One. 2018 Jun 13;13(6):e0198067. doi: 10.1371/journal.pone.0198067 (PMC5999221; doi:10.1371/journal.pone.0198067)

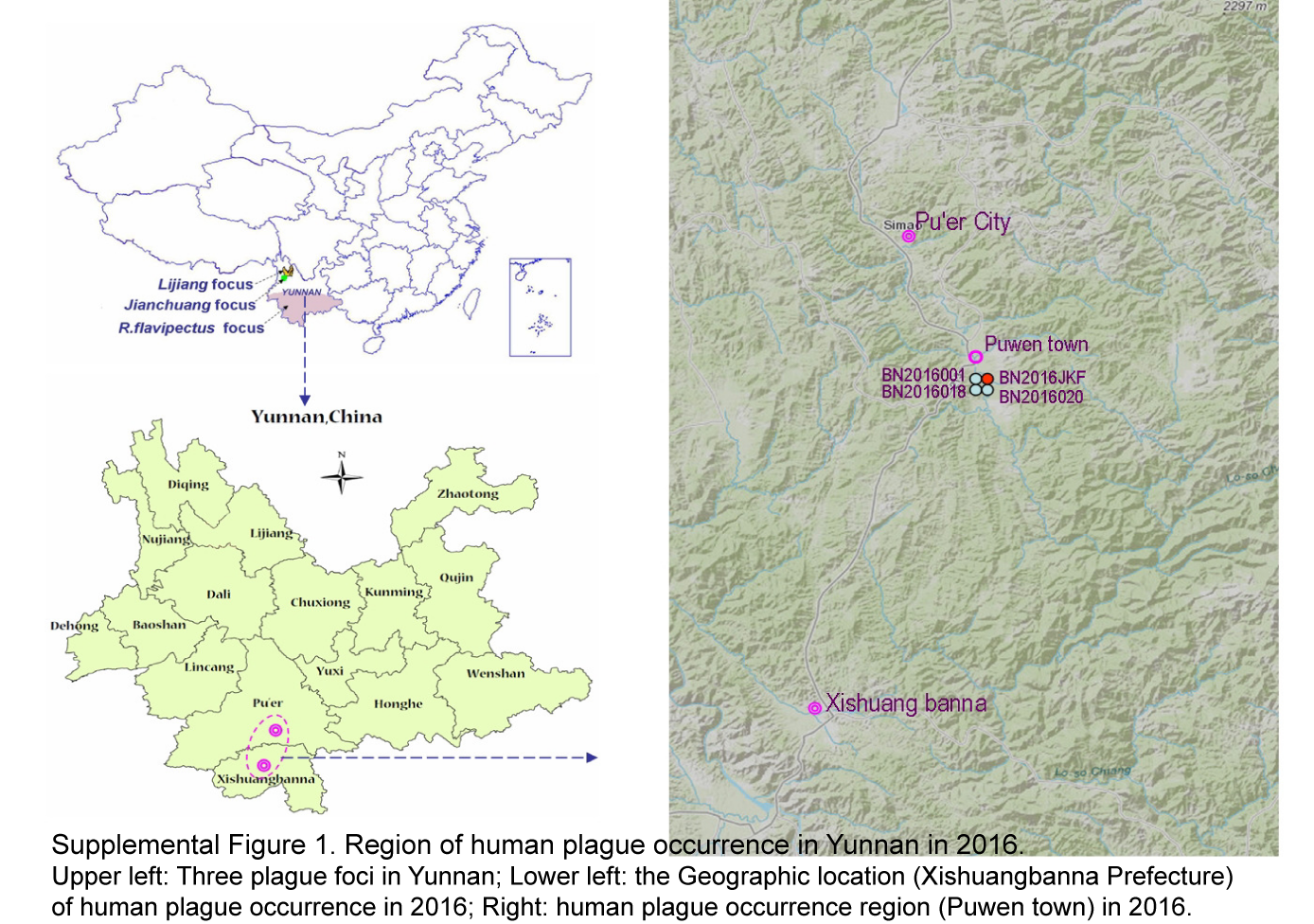

Supplement: S1 Fig — Upper left: Three plague foci in Yunnan; Lower left: the Geographic location (Xishuangbanna Prefecture) of human plague occurrence in 2016; Right: human plague occurrence region (Puwen town) in 2016. (TIF) [file pone.0198067.s001.tif]

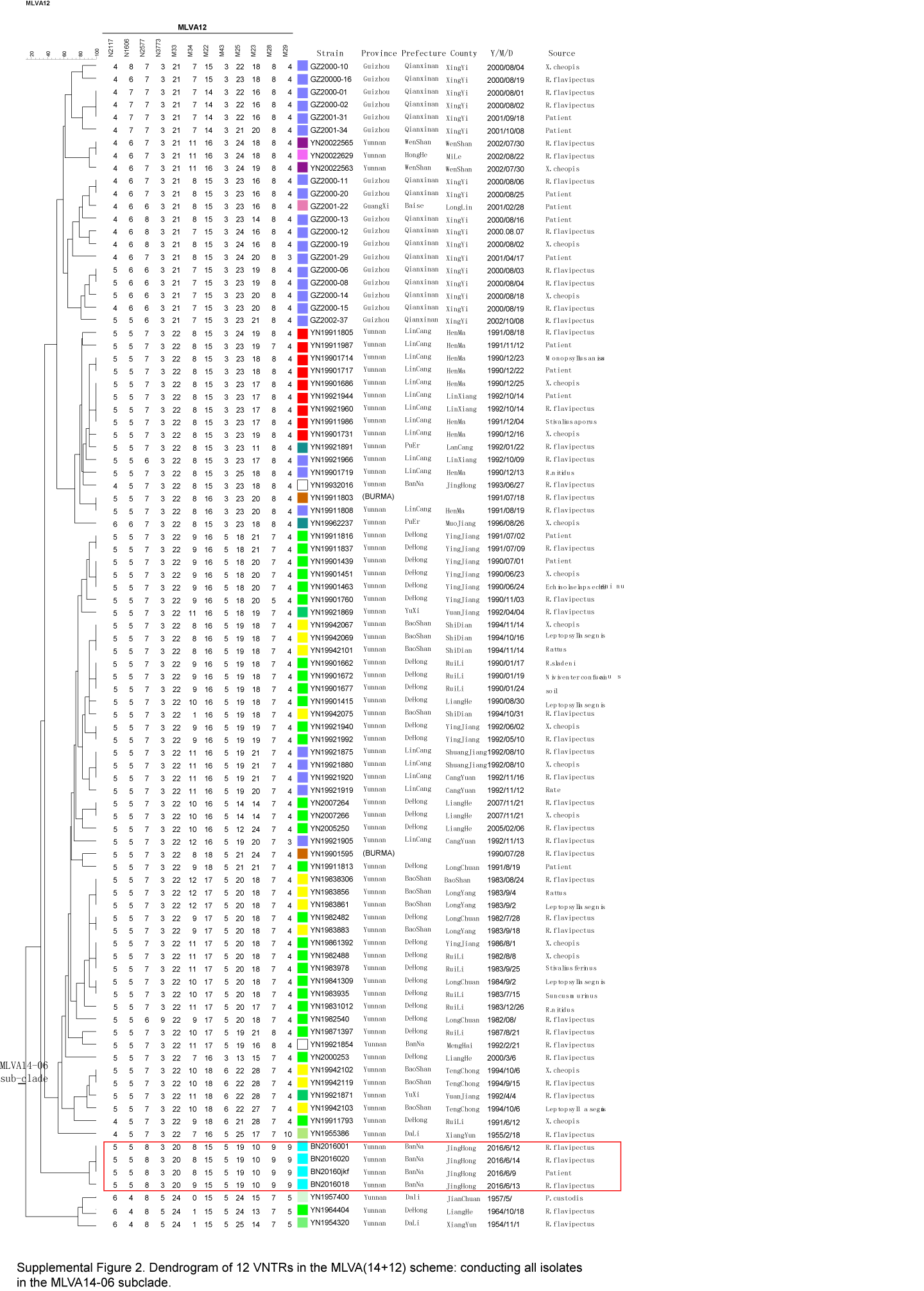

Supplement: S2 Fig — The columns depicted in different colors indicate prefectures in Yunnan or other provinces or strains isolated in Burma (the same strains as depicted in Fig 2). (TIF) [file pone.0198067.s002.tif]
